# Temporal Trends in Treatment Outcomes for HIV-1 and HIV-2-Infected Adults Enrolled in Côte d'Ivoire's National Antiretroviral Therapy Program

**DOI:** 10.1371/journal.pone.0098183

**Published:** 2014-05-27

**Authors:** Andrew F. Auld, Kunomboa A. Ekra, Ray W. Shiraishi, Moise Z. Tuho, Joseph S. Kouakou, Fayama Mohamed, Virginie Ettiègne-Traoré, Jennifer Sabatier, Joseph Essombo, Georgette Adjorlolo-Johnson, Richard Marlink, Tedd V. Ellerbrock

**Affiliations:** 1 Division of Global HIV/AIDS, Centers for Disease Control and Prevention, Atlanta, Georgia, United States of America; 2 Division of Global HIV/AIDS, Centers for Disease Control and Prevention, Abidjan, Côte d'Ivoire; 3 National Program for Medical Care of Persons Living with HIV/AIDS, Ministry of Health, Abidjan, Côte d'Ivoire; 4 Elizabeth Glaser Pediatric AIDS Foundation, Abidjan, Côte d'Ivoire; 5 Department of Economy and Finance, Directorate General of Budget and Finance, Abidjan, Côte d'Ivoire; 6 Elizabeth Glaser Pediatric AIDS Foundation, Los Angeles, California, United States of America; University of Pittsburgh Center for Vaccine Research, United States of America

## Abstract

**Background:**

In Côte d'Ivoire during 2004–2007, numbers of ART enrollees increased from <5,000 to 36,943. Trends in nationally representative ART program outcomes have not yet been reported.

**Methodology/Principal Findings:**

We conducted a retrospective chart review to assess trends in patient characteristics and attrition [death or loss to follow-up (LTFU)] over time, among a nationally representative sample of 3,682 adults (≥15 years) initiating ART during 2004–2007 at 34 health facilities. Among ART enrollees during 2004–2007, median age was 36, the proportion female was 67%, the proportion HIV-2-infected or dually HIV-1&2 reactive was 5%, and median baseline CD4^+^ T-cell (CD4) count was 135 cells/µL. Comparing cohorts initiating ART in 2004 with cohorts initiating ART in 2007, median baseline weight declined from 55 kg to 52 kg (p = 0.008) and the proportion weighing <45 kg increased from 17% to 22% (p = 0.014). During 2004–2007, pharmacy-based estimates of the percentage of new ART enrollees ≥95% adherent to ART declined from 74% to 60% (p = 0.026), and twelve-month retention declined from 86% to 69%, due to increases in 12-month mortality from 2%–4% and LTFU from 12%–28%. In univariate analysis, year of ART initiation was associated with increasing rates of both LTFU and mortality. Controlling for baseline CD4, weight, adherence, and other risk factors, year of ART initiation was still strongly associated with LTFU but not mortality. In multivariate analysis, weight <45 kg and adherence <95% remained strong predictors of LTFU and mortality.

**Conclusions:**

During 2004–2007, increasing prevalence among ART enrollees of measured mortality risk factors, including weight <45 kg and ART adherence <95%, might explain increases in mortality over time. However, the association between later calendar year and increasing LTFU is not explained by risk factors evaluated in this analysis. Undocumented transfers, political instability, and patient dissatisfaction with crowded facilities might explain increasing LTFU.

## Introduction

Similar to other countries in West Africa [1], [2], Côte d'Ivoire faces a dual epidemic of HIV-1 and HIV-2 [3]. Current adult HIV-1 prevalence is estimated at 3% [4], while about 5% of HIV-infected adults are HIV-2 or HIV-1&2 dually reactive [5]. Although the burden of the HIV-2 epidemic is limited [1], [6], antiretroviral therapy (ART) programs need to provide supplies and training for adequate identification and treatment of HIV-2, which differs from that of HIV-1 [7], complicating the program in an already challenging setting [8], where resources are limited and political instability has culminated in two civil wars in the last decade [9].

Despite these challenges, the Ministry of Health (MOH) and international partners, including the United States (U.S) President's Emergency Plan for Relief (PEPFAR) and the Global Fund to Fight AIDS, Tuberculosis, and Malaria (GFATM), have increased numbers of ART enrollees about 20-fold from less than 5,000 to 104,750 during 2004–2012 [10]. Although sub-national ART programs in Côte d'Ivoire have reported their treatment experience for the period 2004–2008 [5], [11], these previous reports cannot be considered nationally representative [12], [13]. Investigating and reporting national ART program outcomes is important to provide a representative assessment of program quality and justify continued funding [14]–[16]. Describing program trends over time at a national level, and assessment of factors associated with national outcomes, can help to identify areas for national program improvement activities [17], [18].

Therefore, in 2009–2010, we conducted a retrospective, cohort study among a nationally representative sample of adult ART patients starting ART during 2004–2007, to describe trends in patient characteristics at ART initiation over time and trends in mortality and loss to follow-up (LTFU).

## Methods

### Ethics Approval

This study was approved by the Ivorian Ethics Review Committee (*Comité National d'Éthique des Sciences de la Vie et de la Santé*), the Institutional Review Board (IRB) of the U.S. Centers for Disease Control and Prevention (CDC), and the Harvard School of Public Health IRB. Patient informed consent was not required as only routine, anonymized, monitoring data were collected and analyzed.

### Eligibility for ART

During 2004–2007, patients were eligible for ART when diagnosed as having World Health Organization (WHO) stage IV, WHO stage III with CD4 counts ≤350/µL, or WHO stage I/II with CD4 counts ≤200/µL [5]. Prescription of co-trimoxazole (CTX) was indicated for all ART patients with CD4 count ≤350/µL.

For HIV-1-infected patients, recommended first-line ART regimens included stavudine (D4T) or zidovudine (AZT) with lamivudine (3TC) and either nevirapine (NVP) or efavirenz (EFV) or, a triple nucleoside reverse transcriptase inhibitor (NRTI) regimen of AZT, 3TC and abacavir (ABC), if one of the non-nucleoside reverse transcriptase inhibitors (NNRTIs) was contra-indicated. For HIV-2-infected or dually reactive patients, recommended first-line therapy was D4T or AZT with 3TC and ritonavir-boosted indinavir (IND/r).

### Patient Monitoring

At ART initiation, monthly for the first 3 months, and quarterly thereafter, standardized MOH-recommended medical records were completed to monitor disease progression or improvement. Patients collected medications monthly from clinic pharmacies where the date of scheduled antiretroviral (ARV) pick-up appointments and actual ARV pick-updates were documented.

### Study Design and Population

This was a retrospective cohort study. Patient-level data were abstracted from standardized, MOH-recommended medical records onto study questionnaires by trained abstractors from November 2009 through March 2010. Only medical records of adult patients, ≥15 years old at ART initiation, who started ART during 2004–2007, were eligible.

### Sample Size

Sample size calculations were performed using Epi Info software (CDC, Epi Info 2008, Version 3.5.1, Atlanta, GA). To achieve a 95% confidence interval (CI) of +2.5% around the estimate for 12-month attrition, assuming a design effect of 1.5 [13], and a conservative (i.e., higher than expected) 12-month attrition percentage of 50% [19], a sample size of ≥2,301 patient records was needed. To meet the needs of a secondary analysis, aimed at assessing site-level predictors of patient outcomes, we aimed to sample 4,000 medical records.

### Sampling

Of 124 ART delivery sites in the country by December 31, 2007, 78 had provided ART to ≥50 adults. Only 833 (2.3%) of all 36,943 adult patients who had received ART by December 31, 2007, were enrolled at sites that had supported <50 patients on ART by this time. To maintain feasibility, 35 (45%) of the 78 eligible sites were randomly selected, using a two-stage sampling strategy. In stage one, the 78 eligible clinics were divided into three strata based on which organization was largely responsible for implementing the ART program at the site (non-governmental organization, MOH, or GFATM through MOH). Within these three strata, sub-strata were created according to site size (number of ART patients ever enrolled). Site size sub-strata were: small (50–250), medium (251–1,000), and large (>1,000). Within each substratum, SAS 9.2 (SAS Institute Inc., Cary, NC) was used to randomly sample facilities using probability-proportional-to-size sampling. Of the selected 35 clinics, 34 agreed to participate.

In stage two, simple random sampling was used to select 4,000 medical records from the 34 selected, consenting facilities. The total number of medical records selected in each sub-stratum was proportional to the number of eligible records in the corresponding sub-stratum in the general adult ART population by 2007.

### Treatment Outcomes

The primary outcomes of interest after ART initiation were documented death and LTFU, and the secondary outcome of interest was the composite outcome of attrition (documented death or LTFU). A patient was considered LTFU if he/she was absent from the health facility in the 90 days preceding data abstraction, and if there was no documentation of death or transferal to another health facility. The date of LTFU was recorded as the date of the most recent visit. Transfers were censored from time-to-event analyses at the date of transfer. Data for time-to-event analysis (i.e. date of ART initiation and date and nature of the final outcome status) were complete.

### Exposure Variables

Patient-level characteristics routinely captured on standard MOH medical records (Table 1) were considered *a priori* risk factors for inclusion in the multivariable models for each of the three outcomes — death, LTFU, and overall attrition. CD4 count and hemoglobin categories [20], [21] and weight categories [22] were chosen based on published precedent. Suitability of the ART regimen was assigned according to published international guidelines [7], [23]. ART adherence during months 0–6 of ART was estimated by timeliness to drug pick-up appointments (i.e., every day late for a pharmacy drug pick-up appointment during months 0–6 of ART was equivalent to one missed day of ART doses) [24]–[26]. Only site size was included as a site-level variable in this analysis [5], [12].

**Table 1:** Demographic and Clinical Characteristics of Adults at ART Initiation in Côte d'Ivoire during 2004–2007.

		All Patients at Enrollment (n = 3,682)						2004 (n = 300)	2005 (n = 898)	2006 (n = 1,243)	2007 (n = 1,241)	P-value*
				Original		Imputed						
		n	N	%/median	IQR/CI	%/median	IQR/CI					
**Age at Enrollment**												
	Median (IQR) year		3682	36	(31–43)	36	(31–43)	36	36	36	36	0.599
**Sex**												
	Female	2,422	3682	67%	(63–70%)	67%	(63–70%)	67%	67%	64%	70%	0.382
**HIV Type**												
	HIV-1	3,464	3,646	95%	(94–96%)	95%	(94–96%)	97%	94%	95%	95%	0.932
	HIV-2	82	3,646	2%	(1–3%)	2%	(2–3%)	1%	3%	2%	3%	
	HIV-1&2	100	3,646	3%	(2–4%)	3%	(2–3%)	2%	4%	3%	2%	
	Missing	36	3,682	1%								
**Marital Status**												
	Civil union|married	1,636	3,268	50%	(46–53%)	50%	(47–54%)	52%	50%	50%	49%	0.510
	Single|widowed	1,632	3,268	50%	(47–54%)	50%	(46–53%)	48%	50%	50%	51%	
	Missing	414	3,682	11%								
**Employment**												
	Employed	1,601	2,601	61%	(56–66%)	59%	(54–64%)	65%	62%	59%	56%	**0.027**
	Student	75	2,601	3%	(1–4%)	2%	(1–4%)	4%	3%	3%	2%	
	Unemployed	925	2,601	37%	(32–41%)	39%	(34–44%)	31%	35%	39%	42%	
	Missing	1,081	3,682	29%								
**TB Treatment Completed Before ART Start**												
	Yes	310	3,682	9%	(4–13%)	9%	(4–13%)	6%	8%	7%	10%	0.387
	No	3,372	3,682	91%	(87–96%)	91%	(87–96%)	94%	92%	93%	90%	
**TB Treatment at ART Start**												
	Yes	182	3,682	6%	(2–9%)	6%	(2–9%)	3%	5%	5%	7%	0.262
	No	3,500	3,682	94%	(91–98%)	94%	(91–98%)	97%	95%	95%	93%	
**WHO Stage**												
	Stage I/II	587	2,581	20%	(1–38%)	20%	(1–40%)	28%	25%	17%	20%	0.267
	Stage III	1,440	2,581	58%	(44–72%)	58%	(43–72%)	53%	57%	60%	56%	
	Stage IV	554	2,581	22%	(12–33%)	22%	(12–31%)	20%	17%	23%	24%	
	Missing	1,101	3,682	30%								
**Weight**												
	<45 kg	625	3,256	20%	(17–23%)	20%	(17–23%)	17%	17%	20%	22%	**0.014**
	45–60 kg	1,823	3,256	56%	(54–58%)	56%	(53–58%)	53%	57%	57%	54%	
	>60 kg	808	3,256	24%	(20–29%)	24%	(20–29%)	30%	26%	23%	24%	
	Median (Kg)		3,256	53	(46–60)	53	(46–60)	55	54	52	52	**0.008**
	Missing	426	3,682	12%								
**CD4 Count**												
	<50 cells/µL	797	3,343	24%	(21–27%)	24%	(21–27%)	26%	25%	24%	23%	0.346
	50-200/µL	1,512	3,343	45%	(43–48%)	45%	(43–48%)	43%	47%	45%	45%	
	201-350/µL	892	3,343	26%	(24–28%)	26%	(24–28%)	27%	25%	27%	27%	
	>350/µL	142	3,343	4%	(3–5%)	4%	(3–5%)	4%	4%	4%	5%	
	Median (IQR)		3,343	135	(54–226)	135	(54–226)	125	125	136	141	0.363
	Missing n (%)	339	3,682	9%								
**Hemoglobin**												
	<8 g/dL	387	3,149	13%	(11–15%)	14%	(11–16%)	10%	16%	13%	13%	0.757
	> = 8 g/dL	2,762	3,149	87%	(85–89%)	86%	(84–89%)	90%	84%	87%	87%	
	Missing n (%)	533	3,682	14%								
**Co-trimoxazole**												
	Prescribed CTX	2,080	3,682	59%	(46–71%)	59%	(46–71%)	45%	64%	59%	57%	0.841
	Not prescribed CTX	1,602	3,682	41%	(29–54%)	41%	(29–54%)	55%	36%	41%	43%	
**Adherence**												
	<95%	526	1,413	33%	(23–42%)	34%	(25–44%)	26%	27%	35%	40%	**0.026**
	≥95%	887	1,413	67%	(58–77%)	66%	(56–75%)	74%	73%	65%	60%	
	Missing n (%)	2,269	3,682	62%								
**Site Size**												
	>1,000	2,147	3,682	51%	(28–74%)	51%	(28–74%)	88%	76%	45%	36%	**0.001**
	≤1,000	1,535	3,682	49%	(26–72%)	49%	(26–72%)	12%	24%	55%	64%	
**Regimen Appropriateness**												
	Optimal	3,078	3,682	92%	(87–95%)	92%	(88–95%)	78%	93%	93%	92%	0.245
	Sub-optimal	283	3,682	8%	(5–13%)	8%	(5–12%)	22%	7%	7%	8%	
	Missing	321	3,682	10%								

Abbreviations: IQR, inter quartile range; CI, confidence interval; TB, tuberculosis; WHO, World Health Organization; CTX, co-trimoxazole.

*P-value derived from regression models including the baseline covariate and year of ART enrollment. Unadjusted logistic regression, ordered logistic regression, and linear regression were used for binary, multi-level, and continuous variables, respectively.

### Analytic Methods

Data were analyzed using SAS 9.2 (SAS Institute Inc., Cary, NC), and STATA 11 (StataCorp, 2009, Stata Statistical Software, Release 11, College Station, TX). The anonymized dataset is available upon request from the analysis working group, comprising the corresponding author, members of the MOH, CDC, and the Elizabeth Glaser Pediatric AIDS Foundation.

Missing data, reported for each baseline covariate of interest in Table 1, were assumed to be missing at random (MAR), and were imputed using multiple imputation with chained equations [27]. The ice [28]–[30] procedure in STATA was used to create 20 imputed datasets for each of the following outcomes: (1) documented death, (3) LTFU, and (3) overall attrition [13]. The imputation model included the event indicator, all study variables, and the Nelson-Aalen estimate of cumulative hazard [31]. For all analyses using imputed data, estimates were combined across the imputed datasets according to Rubin's rules [27] using the mim procedure in STATA [32].

To assess the association between baseline characteristics and year of ART initiation, linear, logistic, ordered, or multinomial logistic regression models, accounting for study design, were used for continuous, binary, ordinal, and nominal categorical variables, respectively. To assess the association between baseline characteristics and sex, unadjusted logistic regression, accounting for study design, was used.

A competing risks model was used to estimate 6-, 12-, and 24-month mortality and LTFU for each annual cohort of adults starting ART during 2004–2007 [33]. Stacked cumulative incidence curves were used to illustrate cumulative probability of death and LTFU over time for each annual cohort of adults starting ART [33].

In time-to-event analysis, Cox proportional hazards regression models that controlled for study design were used to estimate crude and adjusted hazard ratios (AHR) and 95% confidence intervals (CI) for covariates of interest [34]. The proportional hazards assumption was assessed using visual methods and the Grambsch and Therneau test [35]. Kaplan-Meier curves were used to examine cumulative probability of retention (1-attrition) over time stratified by baseline variables.

## Results

### Trends in Patient Characteristics at ART Initiation

Data from medical records of 3,682 eligible, adult ART patients were abstracted and analyzed. Year of ART enrollment for adult ART patients included in the study was 2004, 2005, 2006, and 2007, for 6%, 22%, 36% and 36%, respectively. During 2004–2007, 67% of patients were female, median age was 36 years, most patients (95%) were HIV-1-infected, 2% were HIV-2-infected, and 3% were HIV-1&2 dually reactive. These variables did not change significantly over time.

Overall, 59% of patients reported employment, but the proportion reporting employment declined from 65% to 56% during 2004–2007 (p = 0.027). Most patients had WHO stage III (58%) or IV (22%) with no significant changes over time (Table 1). Median ART enrollment weight was 53 kg, but declined from 55 kg to 52 kg during 2004–2007 (p = 0.008). Similarly, the proportion with very low weight (<45 kg) at ART initiation was 20% overall, but increased from 17% to 22% during 2004–2007 (p = 0.014). During 2004–2007, the proportion with hemoglobin <8 g/dL was 14% and this did not change over time. Median CD4 count overall was 135 cells/µL and did not change significantly over time (p = 0.363).

The proportion of patients prescribed CTX at ART initiation was 59% and this did not change significantly over time. The proportion achieving 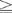
95% adherence to pharmacy pick-up appointments decreased from 74% to 60% during 2004–2007 (p = 0.026). The proportion of patients prescribed sub-optimal ART regimens was 8% and did not change significantly over time (p = 0.245). The proportion of patients enrolling at smaller sites (sites with ≤1,000 enrollees) increased from 12% to 64% (p = 0.001) during 2004–2007.

### Gender Differences

Compared with females at ART enrollment, males had a higher median age (40 vs. 34, p<0.001), a higher prevalence of HIV-2 or dual HIV-1&2 reactivity (8% vs. 4%, p<0.001), and were more likely to report employment (82% vs. 47%, p<0.001). Compared with female ART enrollees, males had a higher median weight (57 kg vs. 50 kg, p<0.001) and a lower prevalence of severe anemia (HB <8 g/dL) (10% vs. 16%, p<0.001), but also a lower median CD4 count (119/µL vs. 146/µL, p<0.001). Adherence to ART <95% was not significantly different between males (33%) and females (35%, p = 0.553).

### ART Regimen Prescription

Eighty-four different initial ART regimens were prescribed to ART enrollees during 2004–2007 (Table 2). Across all patients, D4T+3TC+NVP (44%) and D4T+3TC+EFV (23%), were the most common regimens prescribed.

**Table 2:** Initial ART Regimens for Adult ART Enrollees during 2004–2007 in Côte d'Ivoire.

	Total Patients			HIV-1-Infected			HIV-2-Infected or Dually Reactive		
**Recommended Regimens for HIV-1-Infection but not Recommended for HIV-2 or Dually Reactive Adults**									
	**n**	**N**	**%**	**n**	**N**	**%**	**n**	**N**	**%**
**D4T+3TC+NVP**	1,668	3,682	43.9	1,653	3,500	45.9	15	182	6.6
**D4T+3TC+EFV**	795	3,682	22.7	779	3,500	23.2	16	182	11.8
**AZT+3TC+NVP**	94	3,682	2.6	92	3,500	2.7	2	182	1
**AZT+3TC+EFV**	315	3,682	8.7	306	3,500	8.9	9	182	5.5
**TDF+3TC+EFV**	6	3,682	0.2	6	3,500	0.3	-	182	—
**ABC+3TC+NVP/EFV**	6	3,682	0.2	6	3,500	0.2	-	182	—
**AZT/D4T+3TC+ABC**	137	3,682	2.8	112	3,500	2.4	25	182	11.4
**D4T+ABC+EFV**	2	3,682	0.05	2	3,500	0.06	-	182	—
**Total**	**3,023**	**3,682**	**81.15**	**2,956**	3,500	**83.66**	**67**	182	**36.3**
**Suitable Regimens for HIV-2-Infection or Dually reactive Adults or Second-Line for HIV-1-Infection**									
	**n**	**N**	**%**	**n**	**N**	**%**	**n**	**N**	**%**
**D4T+3TC+LPV/r**	42	3,682	1	9	3,500	0.2	33	182	14.8
**AZT+3TC+LPV/r**	20	3,682	0.4	8	3,500	0.2	12	182	4.8
**ABC+DDI+LPV/r**	1	3,682	0.02	1	3,500	0.02	-	182	—
**ABC+3TC+LPV/r**	1	3,682	0.02	1	3,500	0.02	-	182	—
**ABC+DDI+IND/r**	1	3,682	0.02	1	3,500	0.02	-	182	—
**AZT+3TC+IND/r**	22	3,682	0.7	10	3,500	0.2	12	182	8.2
**D4T+3TC+IND/r**	32	3,682	1	10	3,500	0.3	22	182	12.8
**D4T+3TC+SAQ/r**	2	3,682	0.06	2	3,500	0.07	-	182	—
**TDF+3TC+NFV/r**	1	3,682	0.04	1	3,500	0.04	-	182	—
**Total**	**122**	**3,682**	**3.26**	**43**	3,500	**1.07**	**79**	182	**40.6**
**Regimens which are not Recommended Regardless of HIV-Type**									
	**n**	**N**	**%**	**n**	**N**	**%**	**n**	**N**	**%**
**Monotherapy**	67	3,682	2.3	59	3,500	2.1	8	182	5.8
**Dual Therapy**	43	3,682	1.6	42	3,500	1.7	1	182	0.4
**AZT+D4T combination**	8	3,682	0.3	6	3,500	0.2	2	182	2.4
**2NRTIs + unboosted PI**	70	3,682	1.4	52	3,500	1	18	182	9.5
**ABC+DDI+3TC**	1	3,682	0.03	-	3,500	—	1	182	0.5
**AZT/D4T+3TC+TDF**	4	3,682	0.06	3	3,500	0.05	1	182	0.3
**AZT/D4T+DDI**	18	3,682	0.3	16	3,500	0.3	2	182	0.6
**NRTI+2 NNRTIs**	2	3,682	0.06	2	3,500	0.06	-	182	—
**DDI+3TC+EFV**	1	3,682	0.02	1	3,500	0.02	-	182	—
**DDI+TDF+LPV/r**	1	3,682	0.03	1	3,500	0.03	-	182	—
**NNRTI+PI combination**	1	3,682	0.02	1	3,500	0.02	-	182	—
**Total**	**216**	**3,682**	**6.12**	**183**	3,500	**5.48**	**33**	182	**19.5**
**Summary**									
**Optimal Regimens**	3,078	3,682	91.6%	2,999	3,500	84.7%	79	182	40.5%
* **Sub-optimal Regimens**	283	3,682	8.4%	183	3,500	5.4%	100	182	55.9%
**unknown**	321	3,682	9.5%	318	3,500	9.9%	3	182	3.6%

Abbreviations: D4T, stavudine; 3TC, lamivudine; NVP, nevirapine; EFV, efavirenz; ABC, abacavir; LPV/r, lopinavir-ritonavir; IND/r, indinavir-ritonavir; SAQ/r, saquinavir-ritonavir; NFV/r, nelfinavir-ritonavir; NRTI, nucleoside reverse transcriptase inhibitor; NNRTI, non-nucleoside reverse transcriptase inhibitor; PI, protease inhibitor; DDI, didanosine.

*This includes all regimens in the sub-section “regimens which are not recommended regardless of HIV type” and all regimens in the section “recommended regimens for HIV-1-infection but not recommended for HIV-2 or dually reactive adults” that were prescribed for HIV-2-infected or dually reactive adults.

Among 182 HIV-2-infected or dually reactive adults, optimal first-line therapy of two NRTIs and a boosted PI were prescribed to 41% of patients. Sub-optimal regimens were prescribed to the remaining 56% of HIV-2-infected or dually reactive adults: two NRTIs with an NNRTI were prescribed to 25%, triple NRTIs to 11%, two NRTIs with an unboosted PI to 10%, and monotherapy to 6% (Table 2).

Overall, 283 (8%) of all patients were prescribed sub-optimal regimens (Table 2). Sub-optimal ART regimen prescription was more common for HIV-2-infected or dually reactive patients compared with HIV-1-infected patients (56% vs. 5%, p<0.001).

### Treatment Outcomes

Among 3,682 enrollees, 1,778 (49%) were alive on ART at the same facility by the time of data abstraction, 1,481 (40%) became LTFU, 216 (7%) died, and 207 (6%) had been transferred out to another facility. At 6, 12, 24, 36, 48, and 60 months after ART initiation, ART retention was 79%, 74%, 65%, 56%, 48%, and 46% respectively.

**Figure 1:**
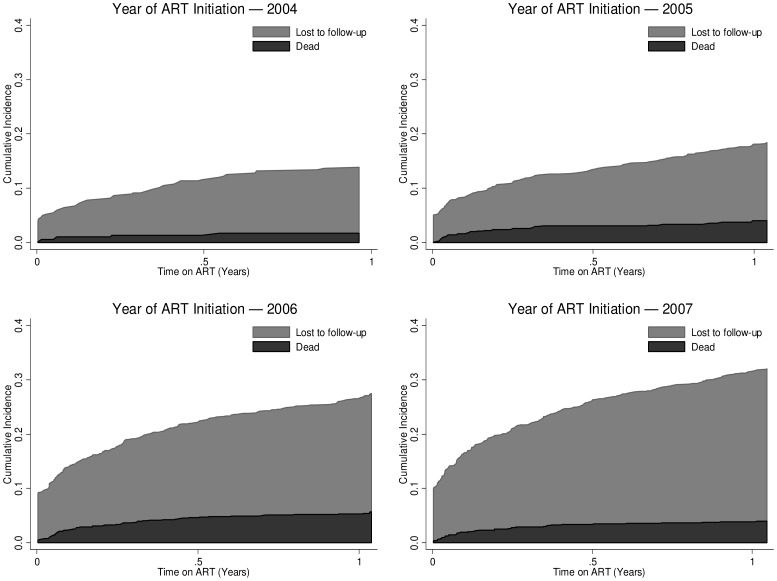
Cumulative Incidence of Mortality and Loss to Follow-up (LTFU) among Adults Enrolled in Côte d'Ivoire's National ART Program during 2004–2007.

During 2004–2007, 12-month retention declined from 86% for 2004 ART enrollees, to 82% for 2005, 73% for 2006, and 69% for 2007 enrollees (Table 3, Figure 1). Declines in 12-month retention were due to increases in 12-month mortality from 2% to 4%, and LTFU from 12% to 28% for 2004 compared with 2007 enrollees. Similarly, rates of mortality increased from 1.5/100 person-years (PY) for 2004 enrollees to 3.9/100 PY for 2007 enrollees, while rates of LTFU increased from 9.2/100 PY for 2004 enrollees to 28.1/100 PY for 2007 enrollees (Table 4). Rates of overall attrition increased from 10.7/100 PY for 2004 enrollees to 32.0/100 PY for 2007 enrollees (Table 5).

**Table 3:** Incidence of Death and Lost to Follow-up among Adult ART Enrollees in Côte d'Ivoire during 2004–2007 by Calendar Year of ART Initiation*.

	Years After ART Initiation	2004	2005	2006	2007
**Death**	**0.5**	1.3%	3.1%	4.7%	3.4%
	**1**	1.8%	4.0%	5.4%	3.9%
	**2**	3.4%	4.8%	6.8%	5.6%
	**3**	4.0%	5.7%	8.0%	7.0%
	**4**	4.2%	7.1%	8.2%	7.0%
					
**LTFU**	**0.5**	10.3%	10.3%	17.5%	22.6%
	**1**	12.1%	14.1%	21.2%	27.6%
	**2**	16.5%	19.8%	27.5%	39.5%
	**3**	21.4%	24.1%	37.6%	49.0%
	**4**	26.6%	32.9%	50.4%	49.0%
					
**Attrition** **	**0.5**	11.6%	13.4%	22.2%	26.1%
	**1**	13.9%	18.1%	26.6%	31.5%
	**2**	20.0%	24.5%	34.3%	45.1%
	**3**	25.3%	29.9%	45.6%	56.0%
	**4**	30.8%	40.0%	58.6%	56.0%
					
**Retention** **	**0.5**	88.4%	86.6%	77.8%	73.9%
**(1-attrition)**	**1**	86.1%	81.9%	73.4%	68.5%
	**2**	80.0%	75.5%	65.7%	54.9%
	**3**	74.7%	70.1%	54.4%	44.0%
	**4**	69.2%	60.0%	41.4%	44.0%

Abbreviations: LTFU, loss to follow-up; ART, antiretroviral therapy.

*Incidence estimates were derived from a competing risks analysis.

**Attrition is defined as the proportion of patients who have died or been loss to follow-up. Retention is defined as (1-attrition). Transfer outs were censored at the time of transfer.

**Table 4:** Predictors of Death and Loss to Follow-up among Adult ART Enrollees in Côte d'Ivoire during 2004–2007.

			Death							LTFU						
			Crude				Adjusted			Crude				Adjusted		
		No	Rate	HR	(95% CI)	p	AHR	(95% CI)	p	Rate	HR	(95% CI)	p	AHR	(95% CI)	p
**Age at enrolment**		3,682	**—**	1.10	(0.96–1.26)	0.154	1.10	(0.92–1.31)	0.267	**—**	**0.93**	**(0.87**–**0.99)**	**0.019**	**0.89**	**(0.83–0.96)**	**0.003**
**Sex**																
	Female	2,422	2.8	1.00	—	—	1.00	—	—	16.0	1.00	—	—	1.00	—	—
	Male	1,260	3.8	1.34	(0.95–1.90)	0.091	**1.65**	**(0.98–2.76)**	**0.058**	20.9	**1.28**	**(1.11–1.46)**	**0.001**	**1.57**	**(1.31–1.89)**	**<0.001**
**HIV Type**																
	HIV-1	3,464	3.1	1.00	—	—	1.00	—	—	17.6	1.00	—	—	1.00	—	—
	HIV-2	82	2.7	0.84	(0.23–3.03)	0.780	1.07	(0.26–4.35)	0.919	21.6	1.23	(0.70–2.15)	0.457	1.25	(0.72–2.17)	0.412
	HIV-1&2	100	2.4	0.79	(0.24–2.58)	0.683	0.81	(0.25–2.62)	0.713	13.0	0.76	(0.54–1.07)	0.109	0.87	(0.63–1.20)	0.379
**Marital Status**																
	Civil union|married	1,636	2.8	1.00	—	—	1.00	—	—	17.3	1.00	—	—	1.00	—	—
	Single|widowed	1,632	3.4	1.18	(0.90–1.54)	0.209	1.22	(0.93–1.59)	0.135	17.8	1.02	(0.88–1.18)	0.790	1.02	(0.89–1.18)	0.743
**Employment**																
	Employed	1,601	2.8	1.00	—	—	1.00	—	—	17.0	1.00	—	—	1.00	—	—
	Student	75	1.7	0.61	(0.10–3.78)	0.581	0.61	(0.08–4.61)	0.614	19.5	1.14	(0.67–1.93)	0.622	1.01	(0.63–1.62)	0.964
	Unemployed	925	3.7	1.31	(0.83–2.07)	0.231	1.27	(0.74–2.20)	0.366	18.3	1.05	(0.84–1.31)	0.643	1.08	(0.87–1.34)	0.465
**Current TB Treatment**																
	No	3,500	3.0	1.00	—	—	1.00	—	—	17.3	1.00	—	—	1.00	—	—
	Yes	182	4.2	1.29	(0.71–2.33)	0.386	0.96	(0.51–1.81)	0.898	23.2	1.27	(0.75–2.14)	0.353	1.23	(0.84–1.80)	0.274
**WHO Stage**																
	Stage I/II	587	1.1	1.00	—	—	1.00	—	—	19.0	1.00	—	—	1.00	—	—
	Stage III	1,440	3.0	2.68	(0.98–7.37)	0.055	1.91	(0.80–4.56)	0.136	15.8	0.82	(0.62–1.10)	0.173	**0.73**	**(0.56–0.96)**	**0.025**
	Stage IV	554	5.7	**4.90**	**(1.73–13.86)**	**0.004**	**3.07**	**(1.22–7.72)**	**0.020**	21.5	1.08	(0.77–1.51)	0.658	0.82	(0.57–1.18)	0.270
**Weight (kg)**																
	>60	808	1.7	1.00	—	—	1.00	—	—	13.6	1.00	—	—	1.00	—	—
	45–60	1,823	3.0	**1.72**	**(1.15–2.57)**	**0.011**	1.40	(0.93–2.11)	0.098	16.9	1.21	(1.00–1.47)	0.054	1.29	(1.00–1.68)	0.053
	<45	625	5.6	**2.97**	**(1.85–4.77)**	**<0.001**	**2.05**	**(1.22–3.46)**	**0.010**	26.6	**1.80**	**(1.46–2.20)**	**<0.001**	**1.90**	**(1.49–2.41)**	**<0.001**
**CD4 Count (cells/µL)**																
	>350	142	1.8	1.00	—	—	1.00	—	—	15.1	1.00	—	—	1.00	—	—
	201–350	892	1.7	0.98	(0.36–2.66)	0.973	1.14	(0.42–3.10)	0.791	16.4	1.09	(0.77–1.54)	0.621	1.20	(0.84–1.70)	0.300
	50–200	1,512	2.9	1.65	(0.63–4.27)	0.290	1.69	(0.64–4.48)	0.274	16.1	1.06	(0.80–1.42)	0.661	1.13	(0.83–1.54)	0.426
	<50	797	5.7	**3.16**	**(1.24–8.05)**	**0.018**	**3.00**	**(1.13–7.95)**	**0.029**	22.9	1.44	(0.97–2.15)	0.070	1.42	(0.95–2.14)	0.086
**Hemoglobin (**g/dL)																
	> = 8	2,762	2.8	1.00	—	—	1.00	—	—	17.2	1.00	—	—	1.00	—	—
	<8	387	5.2	**1.84**	**(1.20–2.83)**	**0.007**	**1.46**	**(0.95–2.24)**	**0.084**	20.3	1.15	(0.84–1.57)	0.383	1.07	(0.80–1.41)	0.638
**Co-trimoxazole**																
	Prescribed CTX	2,080	3.4	1.00	—	—	1.00	—	—	15.0	1.00	—	—	1.00	—	—
	Not prescribed CTX	1,602	2.5	0.72	(0.44–1.18)	0.181	0.73	(0.47–1.13)	0.146	21.5	**1.41**	**(1.04–1.93)**	**0.030**	**1.40**	**(1.12–1.75)**	**0.005**
**Adherence** *																
	≥95% adherent	887	2.1	1.00	—	—	1.00	—	—	14.6	1.00	—	—	1.00	—	—
	<95% adherent	526	5.5	**2.33**	**(1.11–4.87)**	**0.030**	**2.08**	**(0.97–4.46)**	**0.058**	25.1	**1.59**	**(1.10–2.31)**	**0.018**	**1.51**	**(1.12–2.03)**	**0.013**
**Site Size (# adult ART enrollees)**																
	>1,000	2,147	2.0	1.00	—	—	1.00	—	—	14.1	1.00	—	—	1.00	—	—
	≤1,000	1,535	4.7	**2.13**	**(1.03–4.40)**	**0.043**	1.75	(0.89–3.44)	0.099	22.6	1.49	(0.68–3.27)	0.306	1.14	(0.65–1.99)	0.627
**ART Year**																
	2004	300	1.5	1.00	—	—	1.00	—	—	9.2	1.00	—	—	1.00	—	—
	2005	898	2.2	1.39	(0.78–2.50)	0.251	1.12	(0.59–2.14)	0.715	11.0	1.26	(0.95–1.67)	0.101	1.34	(0.98–1.85)	0.068
	2006	1,243	3.7	**2.06**	**(1.06–4.03)**	**0.035**	1.21	(0.53–2.75)	0.633	18.4	**2.22**	**(1.71–2.88)**	**<0.001**	**2.16**	**(1.63–2.88)**	**<0.001**
	2007	1,241	3.9	1.79	(0.89–3.62)	0.099	1.02	(0.42–2.48)	0.962	28.1	**3.15**	**(2.30–4.30)**	**<0.001**	**2.98**	**(2.17–4.09)**	**<0.001**
**Regimen**																
	Appropriate	3,078	3.2	1.00	—	—	1.00	—	—	17.7	1.00	—	—	1.00	—	—
	Sub-optimal	283	1.5	0.50	(0.15–1.71)	0.259	0.65	(0.19–2.20)	0.473	16.0	0.94	(0.70–1.26)	0.660	1.05	(0.79–1.40)	0.724

Abbreviations: LTFU, loss to follow-up; ART, antiretroviral therapy; HR, hazards ratio; CI, 95% confidence interval; AHR, adjusted hazards ratio CTX, co-trimoxazole.

*A high proportion of data were missing for adherence (62%). Therefore, similar to other reports [46], we generated multivariate models with and without this variable, to assess effect on hazards ratios for other covariates, and noted no significant differences.

**Table 5:** Predictors of Attrition among Adult ART Enrollees in Côte d'Ivoire during 2004–2007.

			Crude				Adjusted		
		No	Rate	HR	(95% CI)	p	AHR	(95% CI)	p
**Age at Enrollment**		3,682	**—**	0.95	(0.90-1.01)	0.096	**0.92**	**0.86–0.99**	**0.030**
**Sex**									
	Female	2,422	18.7	1.00	—	—	1.00	—	—
	Male	1,260	24.7	**1.29**	**(1.16–1.43)**		**1.58**	**1.36–1.83**	**<0.001**
**HIV Type**									
	HIV-1	3,464	20.7	1.00	—	—	1.00	—	—
	HIV-2	82	24.4	1.17	(0.66–2.08)	0.578	1.21	0.67–2.21	0.509
	HIV-1&2	100	15.3	0.76	(0.54–1.07)	0.107	0.86	0.65–1.13	0.266
**Marital Status**									
	Civil union|married	1,636	20.1	1.00	—	—	1.00	—	—
	Single|widowed	1,632	21.2	1.04	(0.91–1.19)	0.519	1.05	0.92–1.20	0.454
**Employment**									
	Employed	1,601	19.8	1.00	—	—	1.00	—	—
	Student	75	21.5	1.08	(0.66–1.76)	0.751	0.98	0.63–1.53	0.926
	Unemployed	925	22.0	1.09	(0.88–1.34)	0.423	1.11	0.89–1.37	0.333
**Current TB Treatment**									
	No	3,500	20.2	1.00	—	—	1.00	—	—
	Yes	182	26.7	1.27	(0.82–1.98)	0.271	1.17	0.84–1.65	0.340
**WHO Stage**									
	Stage I/II	587	20.1	1.00	—	—	1.00	—	—
	Stage III	1,440	18.7	0.92	(0.71–1.20)	0.539	0.80	0.63–1.02	0.068
	Stage IV	554	27.2	**1.29**	**(1.00–1.65)**	**0.050**	0.96	0.71–1.29	0.754
**Weight**									
	>60 kg	808	15.3	1.00	—	—	1.00	—	—
	45–60 kg	1,823	20.0	**1.27**	**(1.08–1.51)**	**0.007**	**1.31**	**1.05–1.63**	**0.018**
	<45 kg	625	32.2	**1.93**	**(1.64–2.28)**	**<0.001**	**1.91**	**1.55–2.37**	**<0.001**
**CD4 Count**									
	>350	142	16.8	1.00	—	—	1.00	—	—
	201–350	892	18.2	1.09	(0.79–1.51)	0.572	1.20	0.87–1.66	0.252
	50–200	1,512	19.0	1.13	(0.86–1.49)	0.351	1.19	0.88–1.61	0.251
	<50	797	28.5	**1.63**	**(1.10–2.40)**	**0.016**	1.59	1.05–2.40	0.031
**Hemoglobin**									
	> = 8 g/dL	2,762	20.0	1.00	—	—	1.00	—	—
	<8 g/dL	387	25.5	1.24	(0.94–1.62)	0.116	1.12	0.87–1.46	0.360
**Co-trimoxazole (CTX)**									
	Prescribed CTX	2,080	18.4	1.00	—	—	1.00	—	—
	Not prescribed CTX	1,602	24.0	1.28	(0.95–1.73)	0.094	**1.27**	**1.03–1.57**	**0.028**
**Adherence** *									
	≥95% adherent	887	16.9	1.00	—	—	1.00	—	—
	<95% adherent	526	30.1	**1.65**	**(1.18–2.29)**	**0.006**	**1.54**	**1.15–2.07**	**0.011**
**Site Size**									
	>1,000	2,147	16.1	1.00	—	—	1.00	—	—
	≤1,000	1,535	27.3	1.57	(0.79–3.11)	0.186	1.21	0.73–2.02	0.439
**ART Year**									
	2004	300	10.7	1.00	—	—	1.00	—	—
	2005	898	13.2	1.28	(0.99–1.66)	0.060	**1.32**	**1.00–1.74**	**0.049**
	2006	1,243	22.1	**2.19**	**(1.70–2.81)**	**<0.001**	**2.01**	**1.55–2.60**	**<0.001**
	2007	1,241	32.0	**2.88**	**(2.14–3.89)**	**<0.001**	**2.56**	**1.93–3.40**	**<0.001**
**Regimen**									
	Appropriate	**3,078**	20.9	1.00	—	—	1.00	—	—
	Sub-optimal	**283**	17.5	0.87	(0.63–1.21)	0.390	1.00	0.74–1.33	0.982

Abbreviations: LTFU, loss to follow-up; ART, antiretroviral therapy; HR, hazards ratio; CI, 95% confidence interval; AHR, adjusted hazards ratio CTX, co-trimoxazole.

*A high proportion of data were missing for adherence (62%). Therefore, similar to other reports [46], we generated multivariate models with and without this variable, to assess effect on hazards ratios for other covariates, and noted no significant differences in hazards ratios for other variables in the model.

### Predictors of Outcomes

A 10-year increase in age at ART initiation was associated with an 11% reduction in LTFU risk (AHR 0.89; 95% CI, 0.83–0.96) but not mortality risk (Table 4). Male sex was borderline predictive of mortality (AHR 1.65; 95% CI, 0.98–2.76, p = 0.058) and was associated with LTFU (AHR 1.57; 95% CI, 1.31–1.89). HIV type was not associated with mortality or LTFU risk.

Compared with WHO stage I/II, WHO stage IV was predictive of mortality (AHR 3.07; 95% CI, 1.22–7.72), but not LTFU (Table 4). Compared with having a weight >60 kg, having a weight <45 kg was predictive of mortality (AHR 2.05; 95% CI, 1.22–3.46), and LTFU (AHR 1.90; 95% CI, 1.49–2.41). Compared with having a CD4>350 cells/µL at ART initiation, having a CD4<50 cells/µL was predictive of mortality (AHR 3.00; 95% CI, 1.13–7.95), but not LTFU.

Failure to prescribe CTX was associated with increased risk of LTFU (AHR 1.40; 95% CI, 1.12–1.75), but not documented mortality (Table 4).

Adherence to ART drug refill appointments <95% was associated with borderline increased mortality (AHR 2.08, 95% CI, 0.97–4.46, p = 0.058) and increased LTFU risk (AHR 1.51, 95% CI, 1.12–2.03).

In crude analysis, enrollment at smaller sites (<1,000 enrollees) was predictive of mortality, but this was not significant in multivariable analysis.

In crude analysis, year of ART enrollment was associated with both mortality and LTFU (Table 4). However, in adjusted analysis, year of enrollment was only associated with increasing LTFU rates and not mortality (Table 4).

**Figure 2:**
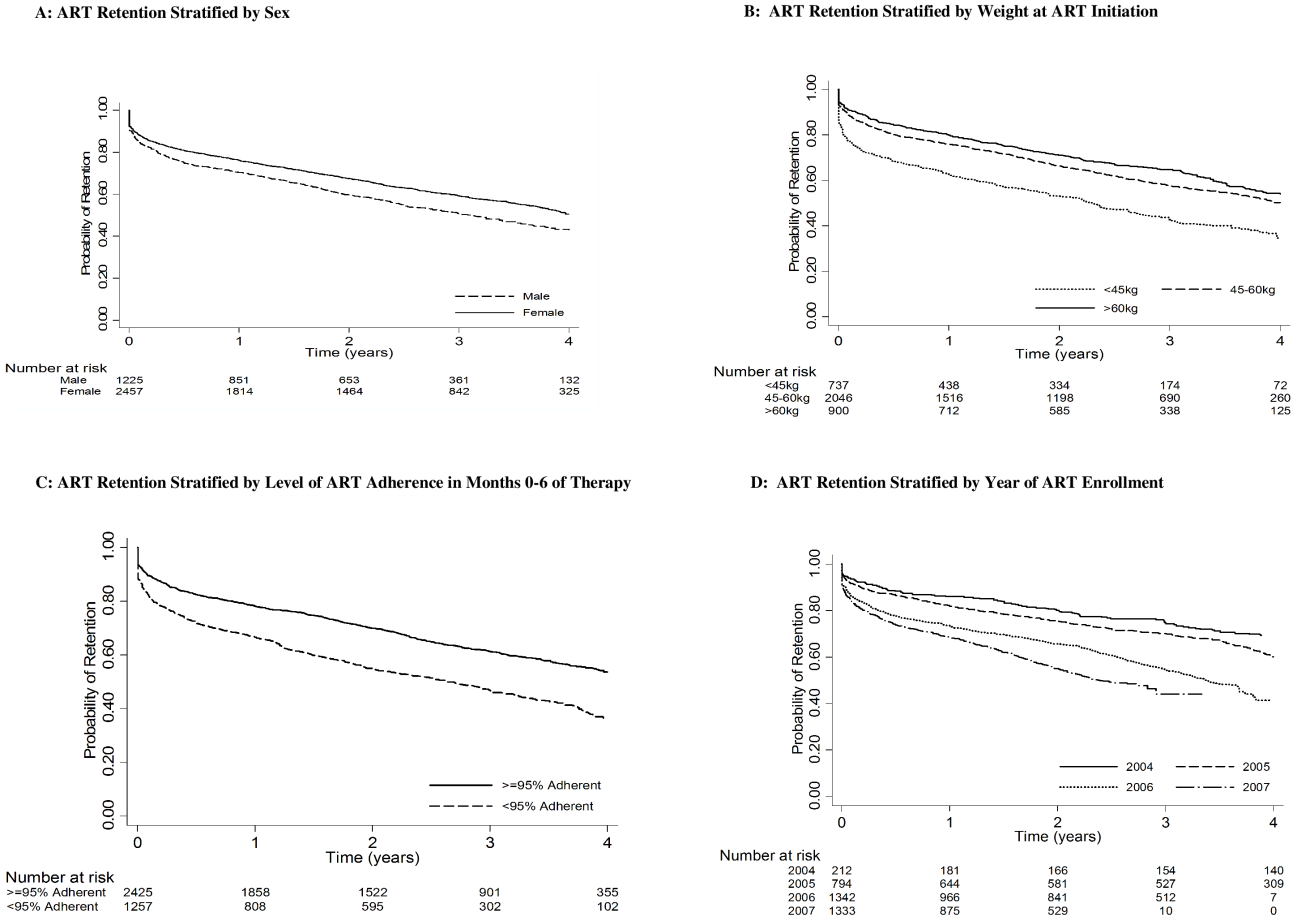
Kaplan-Meier Curves Showing Retention among Adults Initiating ART in Côte d'Ivoire during 2004–2007 Stratified by Risk Factors for Attrition.

Factors predictive of overall attrition in adjusted analysis included younger age, male sex (Figure 2A), very low weight (Figure 2B), CD4 count <50 cells/µL, failure to prescribe CTX at ART initiation, adherence to ART <95% (Figure 2C), and later calendar year of ART initiation (Figure 2 D, Table 5).

## Discussion

This is the first nationally representative evaluation of Côte d'Ivoire's adult ART program, and the first to evaluate trends in program outcomes over time, and has several important findings.

### Declining ART Retention over Time

The most concerning finding of our analysis is the decrease in 12-month retention from 86% for 2004 ART enrollees to 69% for 2007 enrollees. Compared with average 12-month retention for African ART programs during 2004–2007 (75-80% [19], [36]), 12-month retention for ART enrollees in 2006 (72%) and 2007 (69%), was low.

The declining 12-month retention estimates are due to nearly three-fold increases in rates of LTFU (from 9.2–28.1/100 PY), and documented mortality (from 1.5–3.9/100 PY). While year of ART initiation was associated with both mortality and LTFU in unadjusted analysis, in multivariable analysis, controlling for other known predictors of death and LTFU, ART initiation year was only associated with LTFU.

The likely explanation for this finding is that other measured mortality risk factors were confounding the crude association between ART initiation year and mortality. Both prevalence of very low weight at ART initiation and non-adherence to ART in months 0-6 of ART, increased among successive annual cohorts of ART enrollees, and were associated with mortality. In contrast, measured risk factors for LTFU in this study do not explain the association between year of ART initiation and LTFU. This analysis has important implications for the program response to declining retention.

### Program Response to Increasing Mortality

Addressing increasing prevalence of sub-optimal nutritional status and declining ART adherence may help program managers to reverse trends of increasing mortality rates. Increasing prevalence of nutritional insufficiency may be related to increasing food insecurity [37], which may be related to increasing political instability since the late 1990s that culminated in the second Ivorian civil war in 2011 [9]. Alternately, expansion of the ART program to more rural areas, especially in the north of the country, where food insecurity is more common [37], might explain the worsening baseline nutritional status of ART enrollees. The increasing proportion of ART enrollees who report being unemployed (from 31% to 42% during 2004–2007) supports the theory that food insecurity might underpin increasing prevalence of sub-optimal nutritional status. In Côte d'Ivoire, where 23% of the population live on <$1.23/day [38], further research to evaluate the health benefits of integrated nutrition programs in adult ART clinics might be warranted [39]–[41].

Addressing food insecurity, for example through clinic-based food assistance [39]–[41], may also help to address the problem of declining ART adherence [39]. Other interventions to improve adherence might include targeting patients who display sub-optimal pharmacy-based measures of adherence during months 0-6 of ART, with a package of adherence interventions including viral load testing [42], [43]; this targeted approach might improve adherence [43], reduce mortality [44], and reduce LTFU risk [11].

### Program Response to Increasing LTFU

Identifying the causes of increasing LTFU rates in future studies is important to allow identification of possible interventions. Increasing rates of LTFU have been documented in other countries with rapidly expanding ART programs, including South Africa [45], [46] and Mozambique [13]. Two factors may be contributing to increasing LTFU: firstly, with increasing patient load, attention to timely, accurate maintenance of medical records may be compromised, resulting in missing entries for clinic visits or undocumented transfers [46], [47]. Developing and implementing effective electronic monitoring systems, with dedicated data management personnel, could improve data quality and accuracy [47].

Secondly, with increasing patient-to-provider ratios, patient waiting times are increased, and waiting rooms become more crowded [45], [48]–[50]. This may be associated with patient and clinician dissatisfaction with clinic conditions, which may be one cause for increasing LTFU [51]–[55]. Reducing patient-to-provider ratios might be facilitated by several interventions including increasing the workforce, task shifting [56], or decreasing visit frequency for stable patients [57]. One method to decrease visit frequency for stable patients is formation of community adherence support groups (CASG). CASGs comprise groups of 6–10 patients, who take turns to collect the group's ART medications from clinic pharmacies each month. In Mozambique a pilot project significantly reduced 12-month LTFU [58]. Alternately, distribution of ART at locations closer to patient's homes might reduce patient and clinic burden and might improve retention [59].

### Co-trimoxazole for ART Enrollees

In our study, failure to prescribe CTX to ART enrollees was associated with increased LTFU and overall attrition. It is unclear whether CTX reduced morbidity, which contributed to reductions in LTFU [60], [61], or whether clinician compliance with CTX prescription was a marker of higher quality clinical care. Regardless, there is considerable evidence [13], [60], [61] supporting the need to prescribe CTX to all ART enrollees.

### Male Gender

As has been documented in other African cohorts [5], [13], [20], [22], [62], males had a lower baseline median CD4 count than females, a higher risk of LTFU, and marginally increased mortality. Delayed presentation for care might be due to gender norms, which discourage men from admitting ill-health, while higher rates of LTFU might reflect differences in adherence to chronic care [13], [62], [63]. However, higher background mortality among men in general, regardless of HIV status, might explain gender differences in mortality during ART follow-up [62]. In Côte d'Ivoire's general population, mortality is higher among males than females (472 deaths/1,000 men vs. 385 deaths/1,000 women) [64]. Increased male mortality is attributed to accidents, homicide, suicide [65], and increased opportunistic infections [62], [65], [66]. In our cohort, higher male LTFU may also be due to underlying increased mortality [22], a proportion of which goes undocumented [67]. However, further research is needed to inform intervention strategies.

### Younger Age

In our study, as in others [13], [46], younger age was predictive of LTFU risk. Point estimates of LTFU rates were higher in adolescents (aged 15-<20 years at ART initiation) at 19.5/100 PY and young adults (aged 20-<25) at 24.4/100 PY, compared with adults aged 25-<75 at ART initiation (range: 0-18.7/100 PY). This may be because younger people are more mobile. In west Africa, migration for work is particularly common among adults in their twenties and thirties, especially among men [68]. Increased risk for LTFU among adolescents has been documented in other studies [69]. Possible cognitive impairment among perinatally infected children who start ART late as adolescents, lack of youth-friendly services, rigid scheduling, increasing responsibilities, and decreasing involvement of adult caregivers all contribute to the challenge of retaining adolescents and young adults on ART [69]. Youth-specific retention interventions may be needed to keep young adults on ART in Côte d'Ivoire.

### HIV-2 and Dual HIV-1&2 Reactivity

As in other countries [8], HIV-2 and dual reactivity were poorly managed, with 56% of affected patients prescribed sub-optimal first-line regimens. Similar to Burkina Faso [8], 25% of HIV-2-infected or dually reactive patients were prescribed NNRTI-containing regimens, to which HIV-2 is resistant [70]–[72]. A further 11% of patients were prescribed two NRTIs with an unboosted PI, which has been associated with poor treatment outcomes in Côte d'Ivoire [73] and Senegal [7], [74]. Triple NRTI therapy is also not recommended [7], due to poor outcomes [75], [76], and risk of Q151M pan-NRTI resistance [77]. Similarly, mono- and dual-therapy are associated with resistance and poor outcomes [7]. Possible reasons for poor HIV-2 management include insufficient training of clinicians, and low availability of ritonavir-boosted regimens, either due to stock outs [78], or lack of a cold chain prior to availability of heat-stable lopinavir-ritonavir [7]. Clinician training and drug supply and demand issues are being addressed [78].

### Limitations

Firstly, these analyses rely on routinely collected and sometimes incomplete data. Missing data on baseline patient characteristics likely introduced non-differential measurement error. Given the proportion of data missing for the adherence variable, prevalence of non-adherence and hazard ratios associated with non-adherence should be viewed with caution, although findings are in line with other publications from Côte d'Ivoire [11], [39]. Secondly, our reported LTFU rate is likely an over-estimate due to the probability of undocumented death [67] or undocumented transfer [47] being observed as LTFU. Similarly, our reported mortality rate is likely an underestimate of true mortality [67]. Finally, these data show trends for patients enrolled during 2004–2007 and trends may have changed in more recent years.

## Conclusions

Increased prevalence of sub-optimal nutritional status and sub-optimal ART adherence, might explain increases in documented mortality over time. Earlier ART initiation before nutritional compromise and targeted adherence interventions might help reverse trends of increasing mortality. Further research to assess the survival benefit of food supplementation for food-insecure ART enrollees, might be warranted. Increasing LTFU rates are not explained by risk factors analyzed in this report. Undocumented transfers, political instability, or patient dissatisfaction with crowded facilities might explain increasing LTFU. Implementing electronic monitoring systems to improve data quality, and innovative LTFU-prevention strategies, possibly targeting men and younger patients, might reverse trends of increasing LTFU.
